# Development of a core outcome set for orthodontic trials using a mixed-methods approach: protocol for a multicentre study

**DOI:** 10.1186/s13063-017-2098-x

**Published:** 2017-08-04

**Authors:** Aliki Tsichlaki, Kevin O’Brien, Ama Johal, Zoe Z. Marshman, Philip P. Benson, Fiorella B. Colonio Salazar, Padhraig S. Fleming

**Affiliations:** 1 Department of Orthodontics, Barts and the London School of Medicine and Dentistry, Queen Mary University of London, London, E1 1BB UK; 2 Division of Dentistry, Faculty of Biology, Medicine and Health, University of Manchester, Manchester, M13 9PL UK; 3 School of Clinical Dentistry, University of Sheffield, Claremont Crescent, Sheffield, S10 2TA UK; 4 King’s College London Dental Institute, Bessemer Road, London, SE5 9RS UK

**Keywords:** Core outcome set, Orthodontics, Mixed-methods, Delphi

## Abstract

**Background:**

Orthodontic treatment is commonly undertaken in young people, with over 40% of children in the UK needing treatment and currently one third having treatment, at a cost to the National Health Service in England and Wales of £273 million each year. Most current research about orthodontic care does not consider what patients truly feel about, or want, from treatment, and a diverse range of outcomes is being used with little consistency between studies. This study aims to address these problems, using established methodology to develop a core outcome set for use in future clinical trials of orthodontic interventions in children and young people.

**Methods/design:**

This is a mixed-methods study incorporating four distinct stages. The first stage will include a scoping review of the scientific literature to identify primary and secondary outcome measures that have been used in previous orthodontic clinical trials. The second stage will involve qualitative interviews and focus groups with orthodontic patients aged 10 to 16 years to determine what outcomes are important to them. The outcomes elicited from these two stages will inform the third stage of the study in which a long-list of outcomes will be ranked in terms of importance using electronic Delphi surveys involving clinicians and patients. The final stage of the study will involve face-to-face consensus meetings with all stakeholders to discuss and agree on the outcome measures that should be included in the final core outcome set.

**Discussion:**

This research will help to inform patients, parents, clinicians and commissioners about outcomes that are important to young people undergoing orthodontic treatment. Adoption of the core outcome set in future clinical trials of orthodontic treatment will make it easier for results to be compared, contrasted and combined. This should translate into improved decision-making by all stakeholders involved.

**Trial registration:**

The project has been registered on the Core Outcome Measures in Effectiveness Trials (COMET) website, January 2016.

## Background

Orthodontic treatment is carried out, using braces, to correct abnormalities related to the teeth and/or jaw relationships, which are often referred to as a ‘malocclusion’. The most recent Children’s Dental Health Survey in the UK revealed that 35% of 12-year-olds and 28% of 15-year-olds were embarrassed to smile or laugh because of their teeth with 37% and 20% of 12- and 15-year-olds, respectively, having an unmet need for orthodontic treatment [1]. Malocclusion has been linked to bullying and teasing in 13% of 10- to 14-year-olds [2], as well as impaired oral health-related quality of life and social and emotional wellbeing [3]. If not corrected in adolescence, malocclusion may remain a long-term condition that could continue to adversely affect quality of life [4]. By improving appearance and function, orthodontic treatment improves the oral health-related quality of life of young people, particularly in the domains of emotional and social wellbeing [5] and might be considered integral for optimal oral health, where oral health is defined as ‘a standard of health of the oral and related tissues which enables an individual to eat, speak and socialise without active disease, discomfort or embarrassment and which contributes to general wellbeing’ [6].

Orthodontics is currently provided to approximately one third of young people in England and Wales at a cost to the National Health Service (NHS) of £273 million in 2013–2014, accounting for 11.2% of the dental budget [7]. Young people are considered eligible for NHS orthodontic treatment based on an assessment using the Index of Orthodontic Treatment Need (IOTN) [8]. Importantly, the IOTN does not take account of either patient- or parent-important outcomes or the impact of dentofacial anomalies on young peoples’ daily lives.

While the number of clinical trials in biomedical and dental research is increasing, it has been suggested that the reported outcome measures may resonate more with clinicians than with our patients [9]. This is important because the usefulness of a study relies on the outcomes it explores. Concentrating on measures that are solely important to clinicians may result in pertinent issues to patients being overlooked. This situation has been exposed in the field of respiratory medicine with Sinha et al. [10] bemoaning ‘wasted resources or misleading information that overestimates, underestimates or completely misses the potential benefits of an intervention’ secondary to the selection of inappropriate outcomes.

The issues stemming from the use and incomplete reporting of, often inappropriate, outcomes are, therefore, multifaceted. Specifically, the time and resources invested in the research may be wasted with approximately 40 to 89% of published trials being non-replicable due to inadequate descriptions of the interventions and outcomes [11]. Furthermore, accounting for patient values is central to evidence-based medicine, and the integration of these values with clinical research evidence is necessary to enable informed decision-making [12]. Valuable information regarding the effectiveness of an intervention may be overlooked by neglecting to consider outcome measures important to patients. An additional problem is the difficulty in combining the results from studies within systematic reviews. This outcome heterogeneity was exemplified in the Cochrane review of treatment of excessively prominent upper teeth, where the included studies all used allied, but distinct radiographic analyses to answer the same question, precluding meta-analysis [13].

Surprisingly, given the high prevalence of malocclusion and the extensive provision of orthodontic treatment, there is limited information derived from clinical trials to inform decision-making from a patient viewpoint. Specifically, when randomised controlled trials (RCTs) in orthodontics published between 2008 and 2012 were analysed, 42 general outcome measures were identified, with most (63%) focussing on technical or morphological changes that result from treatment, with little reference to values that can be discerned or appreciated by patients [9]. While the previous measures are both necessary and important in assessing the effectiveness of care from a technical perspective, other outcomes of comparable value, such as cost-benefit analyses, adverse effects of treatment, patient perceptions, and the impact of, and compliance with, treatment, have remained largely unexplored. The latter are relevant to patients and provide essential information when operators and patients make shared decisions about care. The findings are comparable to those of Sinha et al. [14], who conducted a systematic review of 159 RCTs concerning children with asthma. They found that short-term disease activity was the most frequently measured outcome domain, with quality of life, functional status and physical consequence of disease being underrepresented.

Recently, work has commenced on the development of an agreed standardized set of outcome measures for health care described as a core outcome set (COS). The Core Outcome Measurement in Effectiveness Trials (COMET) initiative is an international network experienced in the development and promotion of these universally agreed outcome sets across the biomedical field [15]. It is suggested that ‘these outcomes should be measured as a minimum in trials assessing effectiveness of interventions, and would help eliminate issues relating to outcome heterogeneity and reporting bias, while ensuring that wide-ranging perspectives are measured, thus enhancing the value of RCTs and systematic reviews’ [16]. A number of consensus approaches have been used to develop COS, including semistructured and unstructured group discussions, the Delphi approach, consensus development conferences, surveys and nominal group technique [17, 18]. While inclusion of key stakeholders including researchers, clinicians, patients, public, policy-makers and public health professionals is key to developing a meaningful consensus, a recent review has revealed that just 16% of these studies reported public involvement [17]. A search of the COMET database and current literature indicates that COS development has not been undertaken within orthodontics.

In light of the significant cost of orthodontics to the NHS, allied to the lack of agreed outcome measures identified within our previous systematic review [9], the need to develop COS for orthodontic treatment of children and young people is clear. The aim of this study is, therefore, to develop a COS for use in clinical trials involving orthodontic treatment of children and young people. The results will ultimately benefit decision-making by all stakeholders.

## Methods/design

The research methodology follows advice set out by COMET on COS development [15] and is similar to that used in the project that developed a COS for the treatment of otitis media with effusion for children with cleft palate [18]. The main research stages are shown in Table 1.

**Table 1:** Research stages – outline

	Research stage
1	Scoping review
2	Qualitative interviews and focus groups (service users)
3	Delphi surveys (service users and providers)
4	Consensus meeting (service users, providers and policy-makers)

### Stage 1: Scoping review

Scoping reviews are used to map key concepts underpinning a broad research area and are useful for examining emerging evidence [19]. Although scoping reviews follow a systematic approach to evidence gathering, they differ from systematic reviews in that they aim to present a broad overview of evidence relating to a topic, irrespective of study quality, rather than focussing on answering a specific question based on carefully selected study designs [20]. The aim of this scoping review is to identify outcomes employed in contemporary orthodontic research, which will partly be used to inform the long-list of outcomes for the Delphi surveys. The previously published systematic review [9] will thus be updated.

Studies will be eligible if they meet the following inclusion criteria:

- Study design: RCTs and controlled clinical trials (CCTs). All parallel-group trials, including those of cross-over or cluster design, will be considered eligible for inclusion.
- Participants (P): children and young people (aged 18 years or younger) undergoing orthodontic treatment, with no age restrictions.
- Interventions (I): any orthodontic treatment intervention will be included.
- Control (C): any comparison group will be included with no restrictions placed on control groups.
- Outcome measures (O): all reported outcome measures (primary and secondary) will be identified and recorded.
- Exclusion: retrospective studies and laboratory-only based studies will be excluded. Studies involving solely adult patients or patients undergoing orthognathic surgery; patients with cleft lip/palate; obstructive sleep apnoea; syndromic conditions or medical history complications will also be excluded.

#### Search strategy for identification of studies

The electronic search strategy will be updated to include the relevant literature, published from 31 December 2012 to 31 December 2016. The following electronic databases will be searched: MEDLINE via Ovid, EMBASE via Ovid, the Cumulative Index to Nursing and Allied Health Literature (CINAHL) via EBSCO, psycINFO via EBSCO and the Cochrane Central Register of Controlled Trials (CENTRAL) via the Cochrane Library. The search strategy will be informed by the identified PICO concepts and inclusion criteria and tailored to each database to ensure appropriate use of search terms and limits. No language restrictions will be applied and attempts will be made to translate any non-English studies identified. In addition, the reference lists and trials identified in recently published Cochrane systematic reviews will be cross-checked to ensure that no relevant studies are missed.

#### Data extraction

The abstracts of all studies identified by the searches will be assessed by one reviewer with a range of expertise including orthodontics, patient-reported outcome measures and trial design. Full-text reports of studies which meet the inclusion criteria, and of studies for which there is insufficient information in the title and/or abstract to make a clear decision, will be obtained. A second reviewer will help to resolve any uncertainty regarding final inclusion until a consensus is reached.

The primary, and any secondary, outcome measures will be identified from information stated by the authors. If this is not clear, the primary outcome will be inferred from the aim of the study, the sample size calculation, or from the first reported outcome in the results section. Any subsequent outcome measures reported in the results will also be identified and recorded as secondary outcomes. In the event of uncertainty as to which outcomes constitute the primary and secondary outcomes, all will be recorded as primary outcomes and a note will be made in the data extraction sheet, which has previously been piloted. The grouping of outcome measures and outcome domains will be reviewed by the Study Advisory Group (SAG), which will consist of four clinicians with a range of expertise in the fields of orthodontics and patient-centred research as well as two qualitative researchers and a patient representative. The SAG will oversee key stages of the research and will help shape conclusions of the project. The outcome lists will be refined into language that patients understand.

### Stage 2: Focus groups/semistructured interviews with young people

This part of the study will identify outcomes that are important to young people who are about to start, are undergoing or have recently finished, active orthodontic treatment. This qualitative component is necessary to explore perspectives about the benefits and adverse effects of treatment to allow the opportunity to identify the most appropriate outcomes to patients themselves. Focus groups will permit exchange of ideas and views amongst participants, while allowing group agreement on important aspects of malocclusion and orthodontic treatment. Interviews will permit children to define and describe their feelings in their own words and within their own frame of reference. It is anticipated that the focus groups and interviews will provide a depth of information that would not otherwise be achievable through simple questionnaire-based surveys. An iterative approach will be used throughout the qualitative aspects of the research.

#### Inclusion criteria

Young people aged 10 to 16 years of both genders, with a range of dental anomalies before treatment, who are due to commence orthodontic treatment, are currently undergoing treatment or have had their active appliances removed, but are wearing an orthodontic retainer and one or both of their parents/carers.

#### Exclusion criteria

Children and young people with a complicated medical history, cleft lip/palate or syndromes, and those undergoing combined orthodontic-orthognathic surgery treatment.

#### Sampling and recruitment

The research participants will be recruited purposively from orthodontic clinics in primary and secondary care from different areas of England, in London, Manchester and Sheffield. One of the primary care centres is a fully private orthodontic centre, while the other centres offer NHS treatment. The goal of purposive sampling will be to seek participants with a range of malocclusions and a range of views concerning the outcomes of orthodontic treatment. A sufficient number of participants will be enrolled to achieve saturation of information, but not so many as to prohibit detailed analysis. Approximately six to eight focus groups will be conducted, each consisting of three to four participants and around eight young people and parents of children will be interviewed, to achieve a total sample size of approximately 25 to 35 participants based on previous similar research work [18, 21]. However, the exact qualitative sample size may change, as it is estimated pragmatically in order to achieve data saturation; this may, therefore, increase if new opinions are continuing to emerge.

#### Recruitment

Potential participants and their parents will be approached at the clinic at the time of their appointment. A verbal explanation of the study will be provided and they will be given separate written Information Sheets to take away and read. After the initial approach, the young person and their parents will be asked if a researcher may contact them by telephone, after a period of approximately 1 week, with the aim of enquiring if they would be prepared to take part and, if so, arranging a date for the interview/focus group. Written consent will be obtained on the day of the interview. It will be made clear to participants that they can withdraw from the study at any time. Participants attending interviews/focus groups will be given a £10 voucher as a ‘thank you’ token, in accordance with INVOLVE recommendations [22].

#### Data collection

Focus groups and interviews will be arranged at a mutually suitable time and place in a non-clinical area at each centre and plain clothes will be worn by the researchers. Focus groups will be stratified by age group (10–13-year-olds and 14–16-year-olds) and by orthodontic treatment stage (pre-treatment, mid-treatment and post-treatment). They will be conducted with children and with their parents present in the room but not taking part in the discussion. Face-to-face interviews will be conducted with children and parents together. All interviews will be conducted using pseudonyms in order to maintain patient confidentiality at all times and encourage openness of participants. Socioeconomic demographic information will be collected upon completion of consent forms, by recording participants’ postcodes.

#### Topic guide

Focus groups and interviews will be semistructured and based on a topic guide informed by the main research questions and the scoping review, but will aim to cover the major aspects of malocclusion and orthodontic treatment, as experienced by the research participants. The topic guide will first be piloted and updated as necessary. The interviewer will be open to the participants’ narratives and flexible in switching between the interview topics. The interviews and discussions will be recorded using a digital sound recorder and will last between 30 and 60 min. Participants will be allowed to take breaks at any time. All focus groups will be conducted with the assistance of an experienced qualitative researcher, who will be acting as a co-facilitator.

#### Data analysis

Data will be transcribed verbatim following the interviews and will be analysed using Framework Methodology [23] which is appropriate for applied health research [24, 25]. The key steps of Framework Methodology will be followed, namely familiarisation, identification of thematic framework, indexing, charting, mapping and interpretation. The analysis will involve taking the participants’ accounts at face value without imposing any constructs on the views as expressed by the young people and parents. Analysis will occur concurrently with data collection. The chief investigator with the assistance of a qualitative researcher will be involved in the analysis of the qualitative data and in the identification of the range of outcomes of importance to parents and young people to ensure validity of findings. Regular data group meetings will be scheduled with the SAG to enable this. Since the chief investigator has a professional background in orthodontics, the involvement of the SAG and qualitative researchers will encourage reflexive dialogue mitigating against potential bias.

### Stage 3: Delphi surveys

This will involve two 3-round electronic Delphi surveys, one for young people and their parents and the other for relevant clinicians. The lists of outcomes developed from stages 1 and 2 will be cross-referenced and developed into a long-list of items and a response scale designed to allow participants to score their importance using a 9-point scale. The outcomes will be discussed and further refined by the SAG. Each round of the Delphi will be developed and piloted. This method has been used previously in the study developing a COS for the treatment of Otitis Media with Effusion for children with cleft palate (mOMEnt) [26].

#### Clinician sample

The clinician sample will be obtained from the membership lists of specialist orthodontists from the British Orthodontic Society and the General Dental Council. We will approach clinicians on the mailing lists by email and invite them to take part in the surveys, on the condition that they agree and consent to participate in all three rounds.

#### Parents and young people sample

The parents and young people involved will be recruited purposively from the centres previously mentioned and will be different from the participants recruited for the qualitative interviews and focus groups.

#### Delphi surveys

- *Round 1*: participants will be asked to score the importance of each outcome using a 9-point scale, with scores of 1–3 considered ‘not important’, 4–6 ‘important but not critical’ and 7–9 ‘critical’ [27]. There will be free-text fields to allow the participants to add any additional outcomes that they consider to be important. Descriptive statistics will then be generated for each item. If new outcomes are identified from the free-text fields, the SAG will discuss them. All outcomes will be carried over to round 2.
- *Round 2*: participants will be contacted and asked to complete the Delphi again and will be reminded of their score in round 1. They will also be shown the mean scores provided for each outcome by their stakeholder group only. Descriptive statistics will be calculated for each outcome. All outcomes will be carried forward to round 3.
- *Round 3*: participants will be invited to score the outcomes again. In this final round participants will be shown their results as well as the mean scores for each stakeholder group.

For each stage of the Delphi all participants will be asked to complete the online exercise within 4 weeks with reminders sent 2 weeks later. Response rates will be monitored and participant response will be encouraged by repeat emails and telephone reminders. This method achieved adequate response rates in the mOMEnt project [28].

#### Final data analysis

The proportion of each stakeholder group scores will be calculated as 1–3, 4–6 and 7–9. Each outcome score will then be defined as ‘consensus in’ when >70% of participants have scored it as 7–9 and <30% have scored it as 1–3. ‘Consensus out’ will be when >70% of participants have scored an outcome 1–3 and <15% participants have scored it 7–9. All other combinations will be considered to be ‘no consensus’, in accordance with previous research [26, 28].

### Stage 4: Consensus meeting

Finally, consensus meetings will be held with all stakeholders involved including: patients, parents, clinicians and commissioners. The latter will include NHS orthodontic commissioners; these will be apprised of the research at the Delphi stage to facilitate their involvement. In the consensus meeting, the results of each outcome will be presented in turn followed by discussion and re-scoring using an anonymous electronic scoring system. The results of the consensus meeting will be compared to the pre-defined definition of consensus and will be used to produce the final COS. In accordance with COMET recommendations, we ideally expect to have between five and ten outcomes in the final COS [15].

The SAG will have input at all stages of the study. Importantly, they will review the outcomes at each stage and ensure that the wording is appropriate for the stakeholder groups. They will also advise on the structure and content of the Delphi surveys and the final consensus meeting.

#### Research ethics

Ethical approval was obtained from the NHS Health Research Authority (HRA) and the East of England – Cambridgeshire and Hertfordshire Research Ethics Committee (REC reference 16/EE/0466) as well as local R&D approval.

## Discussion

The research process will follow a sequential exploratory design, whereby patient views will be explored with the intent of using this information to further build a quantitative survey instrument [29]. This mixed-methods research design, combining quantitative and qualitative approaches, was selected in order to provide a better understanding of patient experiences and expectations of orthodontic treatment, thus ensuring that the COS and future research outcomes are relevant to the former.

In theory, a simpler method, such as a questionnaire, could be used to gather information from patients, as was the case in the COS development for childhood asthma, which had the advantage of minimising the burden on participants and enabling the involvement of a larger participant sample [30]. However, for the present research, it was felt that it would be best if unprompted patient views and attitudes concerning orthodontic outcomes were obtained, rather than providing quantified answers to preconceived ideas as would be the case with a clinician-derived questionnaire [31]. Additionally, questionnaires do not offer opportunities to clarify ambiguous data and equally there is no means of knowing if participants fully understood or even if they misunderstood the questions posed. Finally, since this COS is directed at children and young people, it was also felt that their opinions would require in-depth analysis in order to be able to subsequently convert these opinions into outcomes. Consequently, it was felt that this research question would best be addressed using an integration of quantitative and qualitative data (i.e. mixed methods). A similar approach was also used in determining key health outcomes and development of a COS for children and young people with neurodisability [32].

At present a diverse range of outcomes is used in orthodontic research, with little or no consistency between studies. This research will lead to a COS for use in future clinical research of orthodontic treatment interventions. It will be challenging to identify the most important outcomes to measure in view of the breadth of this area. Patient involvement is, therefore, central to this study to ensure that patient views are represented, and multiple stakeholder groups will be involved to ensure that the COS is acceptable and adopted in future trials. Development of a COS will establish which outcomes to measure in clinical research, but not how to measure them. It would, therefore, be important for future research to be directed towards developing and validating tools to measure the outcomes included in the final COS, if none already exist.

## Trial status

The scoping review has been completed and we are currently recruiting patients for the qualitative interviews and focus groups.

## Abbreviations

COMET: Core Outcome Measures in Effectiveness Trials
COS: Core outcome set
IOTN: Index of Orthodontic Treatment Need
mOMEnt: Management of Otitis Media with Effusion in Children with Cleft Palate
NHS: National Health Service
RCT: Randomised controlled trials
SAG: Study Advisory Group
