# Erratum to Development of a core outcome set for orthodontic trials using a mixed-methods approach: protocol for a multicentre study

**DOI:** 10.1186/s13063-017-2147-5

**Published:** 2017-09-01

**Authors:** Aliki Tsichlaki, Kevin O’Brien, Ama Johal, Zoe Z. Marshman, Philip P. Benson, Fiorella B. Colonio Salazar, Padhraig S. Fleming

**Affiliations:** 1 Department of Orthodontics, Barts and the London School of Medicine and Dentistry, Queen Mary University of London, London, E1 1BB UK; 2 Division of Dentistry, Faculty of Biology, Medicine and Health, University of Manchester, Manchester, M13 9PL UK; 3 School of Clinical Dentistry, University of Sheffield, Claremont Crescent, Sheffield, S10 2TA UK; 4 King’s College London Dental Institute, Bessemer Road, London, SE5 9RS UK

## Erratum

In the original publication [1] have two author names errors. The original article has been updated to rectify this error. The correct versions of the author names can be found below.

Incorrect:

Zoe Z. Marshman

Philip P. Benson

Correct:

Zoe Marshman

Philip Benson
